# Fraying of Metaphysis in Rickets in an Adolescent Girl

**DOI:** 10.4269/ajtmh.22-0407

**Published:** 2022-10-10

**Authors:** Dinesh Kapil, Ashish Sharma

**Affiliations:** ^1^ Red Cross Hospital, Delhi, India;; ^2^ Rheumatology Clinic, Delhi, India

**Figure 1.**
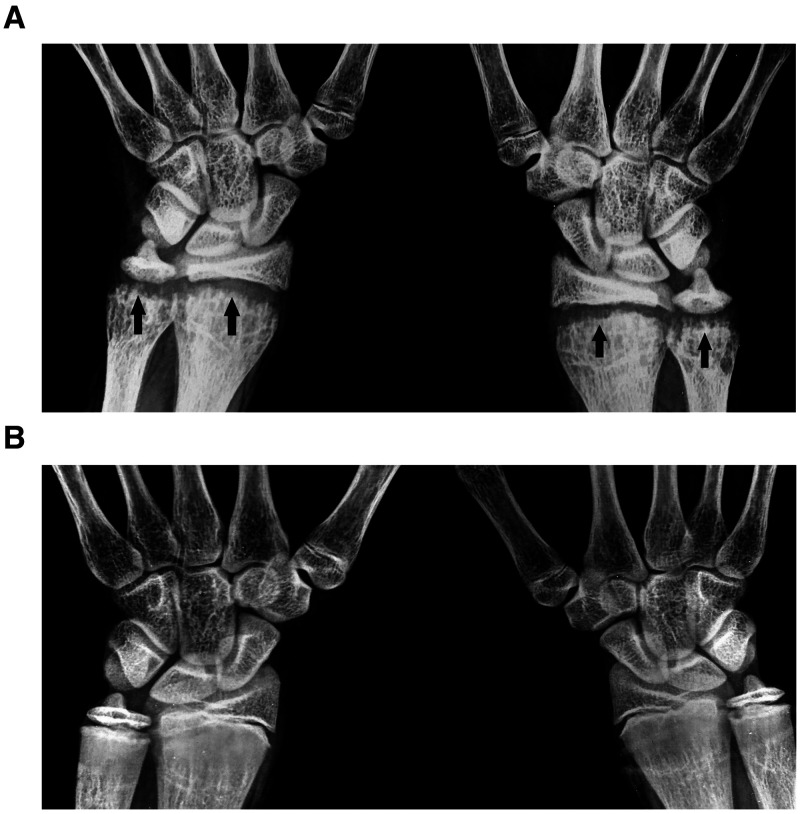
Radiographs of the wrists of the patient with severe rickets. (**A**) Frayed appearance of the distal metaphyses of radius and ulna at presentation (arrows). (**B**) Normalization of the changes, 6 months after treatment.

A 12-year-old girl presented with inability to gain height and difficulty getting up from the floor. She did not have pain in her joints or muscles. Her height was 142 cm and weight 38 kg. Neurological examination was normal, including bulk and strength of muscles of lower limbs. However, broadening of the wrists and “double malleoli” sign were observed. Blood investigations showed 25-hydroxy vitamin D 4.5 ng/mL (normal: 30–50), calcium 8 mg/dL (normal: 8.5–10.5), phosphorus 4.5 mg/dL (normal: 2.5–4.5), and alkaline phosphatase 440 U/L (normal: 45–150). Creatine phosphokinase, erythrocyte sedimentation rate, and C-reactive protein were normal. Radiograph of the wrists showed irregularity of the distal metaphyses of radius and ulna, giving rise to a frayed appearance (Figure 1A). The finding is suggestive of severe rickets. The girl was treated with cholecalciferol and calcium. A repeat radiograph of the hands 6 months later showed marked improvement (Figure 1B).

Rickets is a metabolic bone disease seen in children. It occurs due to the deficiency of calcium and/or phosphate, required for mineralization of the bones. Vitamin D deficiency is a common cause of nutritional rickets in tropical countries.^1^ Mutations of the genes involved in the metabolism of calcium, vitamin D, and phosphorus are rare causes. Rickets is more common in younger children (mean age 1.4 years in a study from New Zealand).^2^ Various skeletal deformities are seen due to suboptimal bone strength. Bowing of legs, widening of wrists, rachitic rosaries, and Harrison sulcus are some of the well-known deformities. However, older children present more often with proximal muscle weakness, fatigue, and pain in the joints and muscles.^3^ Abnormal ossification of the growth plate in growing children is the hallmark of the disease.^1^ It gives rise to frayed appearance of the metaphyses on radiographs.^4^ The finding is more commonly seen in younger children and is unusual at the age of our patient. Reduced outdoor exposure to sunlight due to the prevailing COVID-19 pandemic perhaps resulted in severe vitamin D deficiency. Moreover, fortification of foodstuff with vitamin D is lacking in India.
